# Mitochondrial PARP1 regulates NAD^+^-dependent poly ADP-ribosylation of mitochondrial nucleoids

**DOI:** 10.1038/s12276-022-00894-x

**Published:** 2022-12-06

**Authors:** Jong-Hyuk Lee, Mansoor Hussain, Edward W. Kim, Shang-Jung Cheng, Anthony K. L. Leung, Nima Borhan Fakouri, Deborah L. Croteau, Vilhelm A. Bohr

**Affiliations:** 1 Section on DNA Repair, National Institute on Aging, National Institutes of Health, Baltimore, MD 21224 USA; 2 Department of Biochemistry and Molecular Biology, Bloomberg School of Public Health, Johns Hopkins University, Baltimore, MD 21205 USA; 3 Departments of Oncology, Genetics Medicine, Molecular Biology & Genetics, School of Medicine, Johns Hopkins University, Baltimore, MD 21205 USA; 4 Computational Biology and Genomic Core Facility, National Institute on Aging, National Institutes of Health, Baltimore, MD 21224 USA; 5 Danish Center for Healthy Aging, University of Copenhagen, 2200 Copenhagen, Denmark; 6 Present Address: Department of Biomedical Sciences, Mercer University School of Medicine, Savannah, GA 31404 USA

**Keywords:** Mechanisms of disease, Base excision repair

## Abstract

PARPs play fundamental roles in multiple DNA damage recognition and repair pathways. Persistent nuclear PARP activation causes cellular NAD^+^ depletion and exacerbates cellular aging. However, very little is known about mitochondrial PARP (mtPARP) and poly ADP-ribosylation (PARylation). The existence of mtPARP is controversial, and the biological roles of mtPARP-induced mitochondrial PARylation are unclear. Here, we demonstrate the presence of PARP1 and PARylation in purified mitochondria. The addition of the PARP1 substrate NAD^+^ to isolated mitochondria induced PARylation, which was suppressed by treatment with the inhibitor olaparib. Mitochondrial PARylation was also evaluated by enzymatic labeling of terminal ADP-ribose (ELTA). To further confirm the presence of mtPARP1, we evaluated mitochondrial nucleoid PARylation by ADP ribose-chromatin affinity purification (ADPr-ChAP) and PARP1 chromatin immunoprecipitation (ChIP). We observed that NAD^+^ stimulated PARylation and TFAM occupancy on the mtDNA regulatory region D-loop, inducing mtDNA transcription. These findings suggest that PARP1 is integrally involved in mitochondrial PARylation and that NAD^+^-dependent mtPARP1 activity contributes to mtDNA transcriptional regulation.

## Aging: Key DNA repair protein found in mitochondria

An enzyme critical to healthy DNA repair may help scientists clarify the mechanisms behind mitochondrial dysfunction in aging. As humans age, DNA damage increases, triggering increased activity of the poly [ADP-ribose] polymerase 1 enzyme (PARP1). However, persistent PARP1 activation may be detrimental and could even accelerate aging. PARP1 activity in cell nuclei is well documented, but whether the enzyme is present in mitochondria is disputed. Vilhelm Bohr at the National Institute of Aging, Baltimore, USA, and co-workers conducted experiments on purified mitochondria from human cells and found that mitochondrial PARP1 (mtPARP1) is integral to the mitochondrial matrix and binds to mtDNA. Just as in the cell nucleus, mtPARP1 requires a specific metabolic cofactor for its activity. The mitochondrial enzyme may act as a sensor for this cofactor, regulating signaling for mtDNA transcription.

## Introduction

PARP1 catalyzes the conversion of NAD^+^ to PAR (poly ADP-ribose) and nicotinamide in response to binding to sites of DNA damage^1,2^. PAR is conjugated to histones and other proteins in the vicinity of the DNA lesion and participates in the DNA repair response^3^. Recent studies have shown that PARP1 activation increases with age, possibly reflecting an age-related increase in DNA damage^4^. Although PARP1 is a critical DNA repair enzyme, persistent activation may be detrimental due to intracellular NAD^+^ depletion. Indeed, pharmacological inhibition of PARP1 or supplementation with NAD^+^ precursors extends the lifespan of worms, returns tissue functionality to a more youthful state in mice, and rescues mitochondrial dysfunction in some DNA repair disorders^5,6^. Additionally, it has been reported that PARP1 inhibition increases mitochondrial metabolism^7^, protects mitochondria by reducing the generation of reactive oxygen species (ROS)^8^, improves mitochondrial function^9^, enhances mitochondrial biogenesis, and increases mitochondrial DNA (mtDNA) base excision repair activity^10^.

Most research has focused on understanding the role of PARP1 in the nucleus, but the presence and biological role of mitochondrial PARP1 (mtPARP1) is poorly understood. Although nuclear and mitochondrial ADP ribosylation was recently shown to regulate NAD^+^ balance and dynamics between the nucleus and mitochondria to cope with increased NAD^+^ demand under oxidative stress^11^, the presence and consequences of intramitochondrial PARylation remain unclear^12^.

To systematically investigate the putative presence and biological roles of mtPARP1-mediated PARylation, we performed subcellular fractionation to isolate pure mitochondria from human cells. Ex vivo experiments using purified mitochondria confirmed that NAD^+^ treatment significantly induced mitochondrial PARylation. The induction of mitochondrial PAR by NAD^+^ supplementation was abrogated by treatment with the PARP1 inhibitor olaparib, and mitochondria purified from PARP1-knockout cells showed no increases in PARylation following NAD^+^ or PARP1 inhibitor treatment. Mitochondrial PARylation was evaluated by enzymatic labeling of terminal ADP-ribose (ELTA), which is a newly developed technique for labeling ADP-ribose polymers^13^. Moreover, we directly observed PARP1-dependent PARylation of mitochondrial nucleoids by PAR-specific ADP ribose-chromatin affinity purification (ADPr-ChAP) and PARP1-chromatin immunoprecipitation (ChIP). These results demonstrate that mitochondrial PARylation is mediated by the NAD^+^-dependent activity of mtPARP1. We also found that NAD^+^-induced PAR accumulation facilitated mitochondrial transcription factor A (TFAM) binding to the mtDNA D-loop region, stimulating mtDNA transcription. Taken together, these results directly demonstrate that PARP1 regulates PAR on mitochondrial nucleoids in an NAD^+^-dependent manner.

## Materials and Methods

### Tissue culture

WT and PARP1-KO HeLa cells were generous gifts from Aswin Mangerich^14^. The cells were cultured in Dulbecco’s modified Eagle’s medium (DMEM) supplemented with 10% fetal bovine serum (FBS) and 1% pen-strep and were grown in 20% O_2_/5% CO_2_ at 37 °C. All cell lines used in this study were confirmed to be free of mycoplasma contamination using the LookOut^®^ Mycoplasma qPCR Detection Kit (Millipore-Sigma, St. Louis, MO, USA).

### Mitochondrial purification

Cells were freshly plated on a tissue culture dish 24 h prior to the experiment. The tissue culture media was removed, and the cells were washed with ice-cold mannitol-sucrose (MS) buffer (pH 7.4) (225 mM mannitol, 75 mM sucrose, 5 mM HEPES, 1 mg/ml fatty-acid-free bovine serum albumin (BSA), and 1 mM EGTA). The cells were then scraped with MS buffer containing 100 µM ADP-HPD (Millipore-Sigma) and 5 µM olaparib (Millipore-Sigma). The cell suspensions were homogenized manually using 15 strokes of a Potter-Elvehjem Teflon tissue grinder pestle (Wheaton Science Products, Millville, NJ, USA) in a Dounce homogenizer (Bellco Glass, Vineland, NJ, USA). The homogenate was centrifuged at 2200 × *g* for 3 min at 4 °C. The supernatant was removed and kept on ice, and the pellet was resuspended in MS and centrifuged at 2200 × *g*. The resulting supernatants were pooled and centrifuged at 17,000 × *g* for 10 min. The pellet containing mitochondria was then washed with EGTA-free MS buffer. The mitochondrial pellet was resuspended in EGTA-free MS buffer. Protein concentrations were determined by the standard BCA (Thermo Fisher Scientific, Waltham, MA, USA) method with a BSA standard curve.

### Mitochondrial NAD+ treatment

Isolated mitochondria were resuspended in mitochondrial assay solution (MAS) (70 mM sucrose, 220 mM mannitol, 10 mM KH_2_PO_4_, 5 mM MgCl_2_, 2 mM HEPES, 1 mM EGTA, 5 µM sodium pyruvate and 0.2% (w/v) fatty-acid-free BSA, pH 7.2 at 37 °C). Mitochondrial aliquots were treated with 100 µM NAD^+^ with 5 µM olaparib or vehicle (DMSO) and incubated at 37 °C for 30 min with gentle shaking.

### Mitochondrial outer-membrane stripping

For mitochondrial outer-membrane stripping, mitochondrial isolation was performed using a Qproteome Mitochondria Isolation Kit (Qiagen, Hilden, Düsseldorf Germany). Briefly, HeLa cells were grown asynchronously, and approximately 5 × 10^6^ cells were suspended in lysis buffer, which selectively disrupts the plasma membrane without solubilizing it, resulting in the isolation of cytosolic proteins. Plasma membranes and compartmentalized organelles, such as nuclei, mitochondria, and the endoplasmic reticulum (ER), remained intact and were pelleted by centrifugation at 1000 × *g* for 10 min. The resulting pellet was resuspended in Disruption Buffer, homogenized using a Dounce homogenizer (50 strokes), and centrifuged at 1000 × *g* for 10 min to pellet nuclei, cell debris, and unbroken cells. The supernatant was recentrifuged at 6000 × *g* for 10 min to pellet mitochondria. After removal of the supernatant, mitochondria were washed and resuspended in Mitochondria Storage Buffer. For high-purity preparations, the mitochondria pellet was resuspended in Mitochondria Purification Buffer and carefully pipetted on top of layers of Purification Buffer and Disruption Buffer. The sample was centrifuged at 14,000 × *g* for 15 min. The high-purity mitochondria were pelleted in mitochondria storage buffer. The purified mitochondria were divided into three groups: whole mitochondria, mitochondria treated with 0.1 µg proteinase K, and mitochondria treated with 0.1 µg proteinase K plus 2% Triton X-100 on ice for 30 min. After the reaction, 5 mM PMSF was added to the samples to inhibit proteinase K activity, and the samples were centrifuged at 5000 × *g* for 5 min. The pellet was suspended in NuPAGE LDS sample buffer (Thermo Fisher Scientific) and further analyzed by western blotting.

### Suborganellar mitochondrial fractionation

Suborganellar mitochondrial compartments were isolated as previously described^15,16^. Briefly, isolated mitochondria were resuspended in 0.15 mg/ml digitonin and incubated on ice for 15 min with gentle vortexing every 5 min. The samples were centrifuged at 10,000 × *g*, the resulting supernatant was collected as the outer-membrane fraction, and the pellet was recovered as the mitoplast. To separate the mitochondrial inner membrane and matrix, isolated mitoplasts were resuspended in 100 µl of buffer A and sonicated gently. The samples were then centrifuged at 100,000 × *g* for 30 min at 4 °C. The resulting supernatant was collected as the matrix fraction, and the pellet was recovered as the inner membrane fraction. Ten micrograms of each fraction was used for western blotting.

### Immunoprecipitation

HeLa cells were treated with or without 1 mM NR and NR/olaparib (1 mM/5 µM) for 24 h. After being treated, the cells were lysed with RIPA buffer (Thermo Fisher Scientific) containing protease inhibitors. One milligram of lysate was incubated with 1 µg of TFAM antibody (Proteintech, 22581-1-AP) or normal rabbit IgG for 24 h at 4 °C. Then, 20 µl of magnetic protein A/G beads (Thermo Fisher Scientific) were added to the sample and incubated at 4 °C for 1 h. The beads were washed three times with wash buffer (0.1% NP40 in PBS) on a magnetic stand. NuPAGE LDS dye was added to the beads and boiled for 10 min. Supernatants were loaded on 4–12% NuPAGE gels (Thermo Fisher Scientific) and probed by western blotting.

### Enzymatic labeling of terminal ADP-ribose (ELTA)-based visualization of cellular PAR using gel electrophoresis

ELTA was performed according to a previous publication^13^. PAR was extracted from whole cells or isolated mitochondria. Cellular proteins were precipitated with 20% (w/v) trichloroacetic acid (TCA) on ice for 15 min, centrifuged at 3000 × *g* for 10 min at 4 °C, and washed twice with 70% ethanol. To isolate PAR, 0.5 M KOH was used for to cleave PAR from the modified protein at 37 °C for 1 h and neutralized by 2 M MOPS buffer. Then, DNase (1 mg/mL) and RNaseA (1 mg/mL) were used for nucleic acid digestion at 37 °C for 3 h, followed by Proteinase K (2 mg/mL) treatment overnight for protein digestion. PAR samples were purified by a Monarch RNA cleanup kit (T2040L, New England Biolabs Ipswich, MA, USA). In brief, the samples were first mixed with 2 volumes of binding buffer and then 2 volumes of 95% ethanol. The mixtures were then loaded on the supplied columns, centrifuged, washed twice with wash buffer, and eluted with water. Before ELTA, dot blotting was used to measure the amount of PAR isolated from whole cells and mitochondria (probed by pan-ADP-ribose reagent, Millipore, MABE1016). To compare PAR lengths from samples in an unbiased manner, equal amounts of PAR from whole cells and mitochondria (based on the signal intensity of the dot blot) were mixed with Cy5-dATP, poly(I:C) and OAS1 for ELTA (200 mM Tris-HCl, pH 7.5, 200 mM magnesium acetate, 25 mM DTT) at 37 °C for 2 h. The samples were then purified by a Monarch RNA cleanup kit to remove excess Cy5-dATP. Purified PAR was mixed with 2X PAR loading buffer (50% urea, 20 mM NaCl, 2 mM EDTA) and heated at 70 °C for 10 min before electrophoresis. PAR lengths were then resolved by 15% urea-PAGE.

### Western blotting

Cells were harvested and washed with PBS. Whole-cell lysates (WCL) were solubilized in 1 × western blot sample buffer (12 mM Tris-Cl (pH 6.8), 6% glycerol (v/v), 0.4% SDS, 1% β-mercaptoethanol (v/v), 0.02% bromophenol blue (w/v)) containing a protease inhibitor cocktail (Cell Signaling Technology, Danvers, MA, USA). Purified mitochondria or WCL (1–5 μg) were loaded on Mini-PROTEAN^®^ precast gels (Bio-Rad) for sodium dodecyl sulfate‒polyacrylamide gel electrophoresis (SDS‒PAGE). The proteins were transferred to polyvinylidene difluoride (PVDF) membranes (0.45 μM pore size) using a Mini Trans-Blot^®^ Cell transfer system (Bio-Rad, Hercules, CA, USA). The membranes were blocked in 3% milk in TBST for 30 min at room temperature prior to overnight incubation with primary antibodies at 4 °C. The mitochondrial housekeeping proteins VDAC (ab154856, Abcam, Cambridge, MA, USA) and TOM20 (1802-1-AP, Proteintech) were used to normalize the isolated mitochondrial samples. LMNA (sc71481, Santa Cruz Biotechnology, Dallas, TX, USA) and H3 (ab1791, Abcam) were used as nuclear markers. Tubulin (sc5286, Santa Cruz Biotechnology) was used as a cytosolic marker. Anti-PAR (4336) and PARP1 (39559) were purchased from Trevigen (Gaithersburg, MD, USA). Anti-COX4 (MAB6980, R&D Systems, Minneapolis, MN, USA) and anti-HSP60 (C4810S, Cell Signaling Technology) were used as mitochondrial inner membrane and matrix markers, respectively.

### Chromatin immunoprecipitation (ChIP)

The ChIP assay was performed as previously described^17^ with slight modifications. Briefly, whole cells or isolated mitochondria were cross-linked in a solution of 4% formaldehyde in PBS for 10 min on ice. The cross-linking reaction was stopped by adding glycine to a final concentration of 0.125 M. Mitochondrial pellets were resuspended in buffer B (100 mM Tris–Cl (pH 8.1), 1% sodium dodecyl sulfate (SDS), 10 mM ethylenediaminetetraacetic acid (EDTA), and protease inhibitor cocktail), and the sample was sheared with an S-450 sonicator (Branson, Danbury, CT, USA) or Bioruptor Pico (Diagenode, Denville, NJ, USA). The sonication conditions were optimized by analyzing purified DNA samples on a bioanalyzer (Agilent, DNA1000 Kit). The prepared mtDNA fraction was diluted 1/10 in IP buffer (0.01% SDS, 1.1% Triton X-100, 1.2 mM EDTA, 16.7 mM Tris–Cl (pH 8.1), 167 mM NaCl and a protease inhibitor cocktail) and incubated with PARP1 (39559, Active Motif, Carlsbad, CA, USA), PARP1-Trap (Xta-20, Chromotek, Plannegg, Germany) or TFAM (ABE483, Millipore-Sigma) antibodies overnight at 4 °C. The samples were incubated for 1 h at 4 °C with protein A or G beads. Then, the beads were washed with TSE150 (0.1% SDS, 1% Triton X-100, 2 mM EDTA, 20 mM Tris–Cl (pH 8.1), 150 mM NaCl), TSE500 (0.1% SDS, 1% Triton X-100, 2 mM EDTA, 20 mM Tris–Cl (pH 8.1), 500 mM NaCl) and Buffer III (0.25 M LiCl, 1% NP-40, 1% sodium deoxycholate, 1 mM EDTA, 10 mM Tris-Cl (pH 8.1)) and twice with TE (pH 8.0) for 10 min each. Bead-bound chromatin was eluted with elution buffer (1% SDS, 0.1 M NaHCO_3_ (pH 8.0)) for 30 min at 65 °C. The supernatant containing the chromatin without the beads was isolated and incubated overnight at 65 °C with 200 mM NaCl to reverse the cross-links. Five hundred microliters of the sample was incubated at 50 °C after 10 μl of 0.5 M EDTA, 20 μl of 1 M Tris (pH 6.5) and 4 μl of Proteinase K (20 mg ml^−1^) were added and then purified with phenol/chloroform/isoamyl alcohol. Nucleic acids were precipitated by centrifugation for 30 min at 4 °C after the sample was mixed with 1 μl of glycogen solution (20 mg ml^−1^), 20 μl of 5 M NaCl and 500 μl of isopropanol. The purified nucleic acid pellets were washed with 70% ethanol, dried and dissolved in nuclease-free water.

### ADPr-chromatin affinity purification (ADPr-ChAP)

ADPr-ChAP was performed in the same manner as ChIP, except WWE affinity resin (Tulip Biolabs, Lansdale, PA, USA) was used instead of the antibody and protein A/G beads, as previously described^17^.

### ADPr-ChAP and ChIP sequencing

Cells were ChIPed with anti-TFAM antibody and ChAPed with WWE affinity resin. DNA fragments were ligated to a pair of adaptors for sequencing on an Illumina Hiseq-2000. The ligation products were size-fractionated to obtain 200–300-bp fragments on a 2% agarose gel and PCR-amplified for 18 cycles. Each library was diluted to 8 pM for 76 cycles of single-read sequencing on an Illumina HiSeq-2000 according to the manufacturer’s recommended protocol.

### ADPr-ChAP and ChIP-sequencing data analysis

The sequencing data were uploaded to the Galaxy web platform^18^, and we used the public server at usegalaxy.org to analyze the data. The sequencing reads were mapped against the human genome (GRCh38/hg38) using Bowtie for Illumina^19^ with default parameters. The SAM format outputs were sorted by genomic coordinates, and uniquely mapped reliable reads were used in the subsequent steps. SAM files were preprocessed using SAMtools^20^. We used the MACS tool^21^ to select regions that were enriched for TFAM and PAR. We applied the default settings and found significant regions (P value ≤ 10^−5^) using non-IP chromatin as a control to eliminate nonspecific signals. Unique reads from each dataset were mapped onto the human mtDNA reference sequence (NCBI accession: NC_012920) using BWA. The sequencing reads were aligned on reference coordinates using seqMINER v1.3.3^22^. Integrative Genomics Viewer^23^ was used to visualize the sequencing signal distribution.

### Mitochondrial transcription assessment

After NAD^+^ or vehicle treatment, purified mitochondria were treated with 100 µg/ml RNase A on ice for 30 min to degrade RNA that may have copurified and been on the outside of the mitochondria. Mitochondrial RNA was purified using a miRNeasy Mini Kit (Qiagen). The total mitochondrial RNA concentration was determined and was reverse-transcribed (SuperScript™ IV First-Strand Synthesis System, Thermo Fisher Scientific).

### Quantitative real-time PCR (qPCR) analysis of relative mitochondrial RNA levels and ChIP products

Reverse-transcribed mitochondrial RNA and antibody-bound chromatin obtained by ChIP were quantified by real-time qPCR with a DyNAmo HS SYBR Green qPCR Kit (F-410L, ThermoFisher Scientific) on an iQ5 and CFX Connect Real-time PCR Detection System (Bio-Rad) and then normalized to GAPDH or 1% input chromatin using the 2^−ΔΔCT^ method. The sequences of the primers are listed in Supplementary Table 1.

### Cellular oxygen consumption

Oxygen consumption rate (OCR) measurements were performed using a Seahorse XF­e96 analyzer (Agilent Technologies, Lexington, MA, USA). The cells (2 × 10^4^) were seeded on a Seahorse tissue culture plate overnight, and the following morning, the media was replaced with freshly prepared unbuffered XF assay media at pH 7.4 (Agilent Technologies) supplemented with 11 mM glucose (Millipore-Sigma), 1 mM sodium pyruvate and 1 mM L-glutamate (Thermo Fisher Scientific). The cells were incubated for 1 h at 37 °C in ambient O_2_ and without CO_2_ before measurements were performed. Respiration was measured in 3 blocks. First, the rate of basal respiration was measured with 4× mix (2 min) measure (4 min) cycles. Next, oligomycin (Cf: 2 μM) (Millipore-Sigma) was injected into injection port A to inhibit complex V and assess proton leak-mediated respiration in 3× mix (2 min) measure (4 min) cycles. Then, FCCP (Millipore-Sigma), a proton ionophore (Cf: 1 μM), was added to elicit the maximal uncoupled respiration rate for 3× mix (3 min) measure (3 min) cycles. Next, the complex III inhibitor antimycin A (Cf: 2 μM) plus the complex I inhibitor rotenone (Cf: 500 nM) (Millipore-Sigma) was added to assess nonmitochondrial respiration in 3× mix (2 min) measure (4 min) cycles. For the final injection, Hoechst 33342 dye was injected via port D, and the cells were incubated for 15 min with 5% CO_2_. The cells in each well were counted using an automated cell counter (Celigo Image Cytometer, Nexcelom Bioscience, Lawrence, MA, USA). The OCR values were normalized to the cell number in each well.

### Quantification and statistical analysis

The data are presented as the means ± standard deviation, and p values were calculated as described in the corresponding figure legends. A *p*-value ≤ 0.05 was considered statistically significant. All data are representative of at least three independent experiments. Western blot images were imported and analyzed using FIJI-ImageJ.

## Results

### PARP1 is present in purified mitochondria

**Fig. 1:**
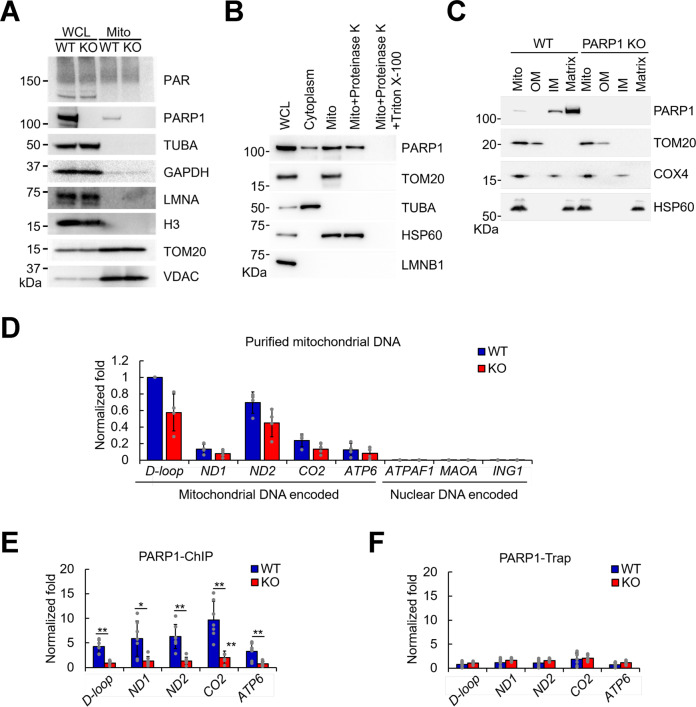
PARP1 is present in purified mitochondria. **a** Purified mitochondria from wild-type (WT) and PARP1-knockout (KO) HeLa cells and whole-cell lysates (WCL) were prepared. Mitochondrial quality was assessed by probing western blots with the indicated antibodies. **b** Purified cytosolic fractions, mitochondrial fractions, and WCLs from WT HeLa cells were prepared. Mitochondrial preparations were treated with proteinase K without or with Triton X-100 on ice for 30 min to strip the mitochondrial outer membrane or mitochondrial matrix proteins, respectively. **c** Mitochondria were purified from WT or PARP1-KO HeLa cells, and suborganellar mitochondrial compartments were isolated. Intact mitochondria (Mito), outer membrane (OM), inner membrane (IM), and matrix fractions were isolated and analyzed by western blotting with the indicated antibodies. **d** Input mitochondrial DNA quality evaluation. Mitochondria were purified from WT and PARP1-KO cells, and 1% of the mitochondrial DNA for the PARP1-ChIP reaction mixture was subjected to real-time PCR using the indicated mitochondrial or nuclear DNA-specific primers (*n* = 4). **e** Mitochondria were purified from WT or KO HeLa cells, and PARP1-ChIP was performed using an antibody that detects the N-terminal half of PARP1 (*n* = 7). Separate two-sample t tests were conducted, and then a false discovery rate (FDR) approach was used to adjust the *p* values for multiple testing. **f** PARP1-ChIP was performed using an antibody that binds to the PARP1 DNA-binding domain. ChIP-purified mitochondrial DNA was subjected to qPCR analysis of the indicated mitochondrial genomic loci (*n* = 7). Error bars represent the standard deviation. *, <0.05; **, <0.01; ***, <0.001.

To evaluate the presence of mitochondrial PAR, we isolated highly purified mitochondria from HeLa cells. The mitochondrial fractions from wild-type (WT) and PARP1-knockout (KO) HeLa cells, which have been shown previously to be completely devoid of PARP1^14^, were enriched for the mitochondrial outer-membrane protein voltage-dependent anion-selective channel protein (VDAC) and translocase of outer mitochondrial membrane 20 (TOM20) and lacked signals for the nuclear protein core histone H3 and lamin A (LMNA) proteins, indicating pure mitochondrial fractions without nuclear contamination (Fig. 1a). Additionally, the mitochondrial fractions showed a complete absence of alpha tubulin (TUBA), suggesting that they were free from cytosolic contamination. Of note, GAPDH localizes to the nucleus, cytosol, and mitochondria and can be used as a control for equal loading. We compared the subcellular compartments and found that PARP1 was most abundant in the nucleus, expressed at intermediate levels in mitochondria and scarce in the cytoplasm (Supplementary Fig. 1). Importantly, we detected PARP1 in mitochondrial samples isolated from WT cells (mtPARP1) but not PARP1-KO cells (Fig. 1a).

To verify PARP1 localization to mitochondria and to check for possible outer mitochondrial membrane contamination from cytosolic PARP1, we stripped the mitochondrial outer membrane with proteinase K (Fig. 1b). The lack of LMNB1 and TUBA and the enrichment of TOM20 confirmed that the mitochondrial fractions were pure (Fig. 1b, 3rd lane). PARP1 in the mitochondrial fraction was also visible under these conditions (Fig. 1b, 3rd lane of the PARP1 blot). The absence of TOM20 and the presence of the HSP60 signal after proteinase K treatment indicated complete disruption of the outer mitochondrial membrane while maintaining an intact mitochondrial matrix (Fig. 1b, 4th lane). Notably, the PARP1 signal remained under these conditions, indicating that PARP1 was present inside the mitochondrial matrix (Fig. 1b, 4th lane of the PARP1 blot). We further performed suborganellar mitochondrial fractionation to isolate the mitochondrial outer membrane (OM), inner membrane (IM) and matrix. The absence of COX4 and TOM20 from the OM and IM fractions shows that each compartment was free of cross-contamination (Fig. 1c, 2nd and 3rd lanes of the TOM20 and COX4 blots). Each membrane fraction was also free of matrix contamination as determined by the absence of HSP60 (2nd and 3rd lanes of the HSP60 blot). Under these conditions, the PARP1 signal was completely absent from the OM but was present in the IM (2nd and 3rd lane of the PARP1 blot). We observed that most of the PARP1 signal originated from the matrix compartment (4th lane of the PARP1 blot). Our findings suggest that the PARP1 signal in the mitochondrial fraction (Fig. 1a) did not originate from cytosolic or nuclear PARP1 contamination of the mitochondrial outer membrane but mostly originated from within the mitochondrial matrix.

As chromatin is one of the major substrates for PARP1 in the nucleus, we evaluated whether the mitochondrial samples had nuclear DNA contamination. We checked the levels of mitochondrial DNA-encoded genes dispersed throughout the mitochondrial genome, such as NADH-ubiquinone oxidoreductase chains 1 and 2 (ND1 and ND2), cytochrome c oxidase subunit 2 (CO2), and ATP synthase subunit A (ATP6), as well as the mtDNA regulatory region (D-loop) and nuclear DNA-encoded genes, such as ATP synthase mitochondrial F1 complex assembly factor 1 (ATPAF1), amine oxidase A (MAOA), and inhibitor of growth protein 1 (ING1), which are markers for the respective subcellular compartments according to previous studies^24^. These regions are also relevant to important mitochondrial functions. Direct real-time PCR analysis of purified mitochondrial samples prepared for PARP1-ChIP verified that the mitochondrial fractions were pure and almost free of nuclear DNA contamination (Fig. 1d). To estimate the amount of mtDNA over nuclear DNA-encoded genes, we analyzed nuclear DNA contamination before and after mitochondrial isolation by calculating the average real-time PCR cycle threshold (Ct) of mitochondrial and nuclear DNA-encoded genes and compared the differences between whole cells and purified mitochondria. Many of the PCRs from purified mitochondria showed significantly delayed nuclear gene amplification cycles compared to those from whole-cell lysates (Supplementary Fig. 2a). In these samples, the Ct values were undetermined within the PCR amplification range (45 cycles). Thus, we considered these values to be 45 to make a conservative estimation. Overall, we found ~40 times less nuclear DNA contamination in the mitochondrial fractions (Supplementary Fig. 2b) than in whole-cell lysates. Based on these data, we believe that the mitochondrial preparations were pure and devoid of nuclear DNA contamination.

As PARP1 interacts closely with DNA/chromatin and makes direct contact with DNA^25^ in the nucleus, we examined whether mtPARP1 could directly bind to mtDNA. We performed PARP1-ChIP using a polyclonal antibody that specifically binds to the N-terminal half of the PARP1 protein. The data are expressed as the PARP1-ChIP signal relative to the nonimmunoprecipitated DNA (input). We found significant PARP1 occupancy throughout the mitochondrial genomic loci in WT cells but not in PARP-1 KO cells (Fig. 1e). PARP1-Trap is a PARP1 affinity resin for the immunoprecipitation of PARP1 that uses an antibody that binds specifically to the DNA-binding domain of PARP1. Real-time PCR analysis of PARP1-Trap-purified DNA failed to detect PARP1 on mtDNA (Fig. 1f). As the epitope is the DNA-binding interface on PARP1, it is likely that the antibody used in these assays prevented free PARP1 from binding to DNA and was also unable to detect PARP1 that was already directly bound to DNA. Together with the PARP1-ChIP data, this result confirms that PARP1 directly binds to mtDNA and not through an indirect secondary interaction with other mtDNA-associated proteins.

### Mitochondrial PARylation is NAD^+^-dependent

**Fig. 2:**
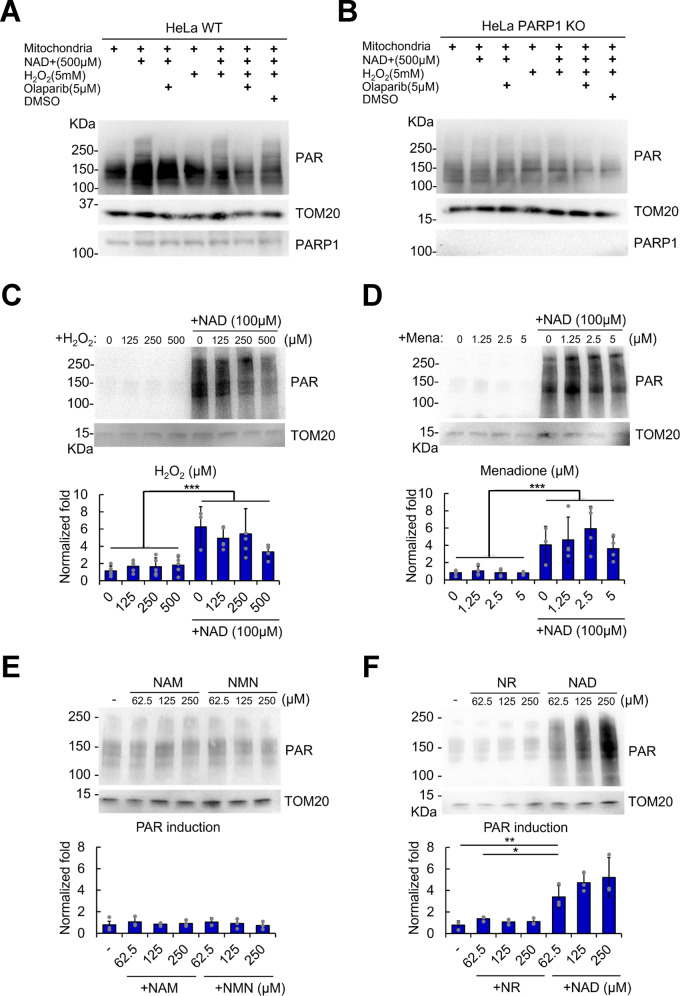
NAD^+^ stimulates mitochondrial PAR via PARP1. Mitochondria purified from HeLa WT (**a**) and PARP1-KO (**b**) cells were treated with the indicated combinations of reagents for 30 min at 37 °C, and the western blots were probed with the indicated antibodies. Mitochondria purified from wild-type HeLa cells were treated with the indicated concentration of H_2_O_2_ (**c**) or menadione (**d**) with/without NAD^+^ for 30 min at 37 °C, and the western blots were probed with antibodies as shown. **e**, **f** Purified mitochondria from HeLa WT cells were incubated with the indicated concentrations of NAD^+^ precursors for 30 min at 37 °C, and the western blots were probed with the indicated antibodies. Blot quantification data are shown. Two-way ANOVA was used to calculate the *p* value (H_2_O_2_, *n* = 5; menadione, *n* = 4; NAM/NMN and NR/NAD, *n* = 3). Error bars represent the standard deviation. ***, <0.001.

Since NAD^+^ is a well-established substrate for PARP1-mediated nuclear PARylation, we sought to determine whether it could similarly modulate mitochondrial PARylation. In purified mitochondria from WT cells, NAD^+^ treatment stimulated PARylation (Fig. 2a). However, olaparib treatment diminished NAD^+^-induced mitochondrial PARylation, suggesting that enzymes inhibited by olaparib were responsible for mitochondrial PARylation after NAD+ treatment. Consistent with the possible role of PARP1 in mitochondrial PARylation, olaparib is a PARP1/2/3 inhibitor used in clinical trials that harbors a nicotinamide moiety that competes with NAD^+^^25–28^. We also tested whether DNA damage induced by hydrogen peroxide (H_2_O_2_) could induce mitochondrial PARylation, but it did not induce PAR, as it does in the nucleus of intact cells^29,30^ (Supplementary Fig. 3a). To test whether this observation was cell-type specific, we also evaluated purified mitochondria from U2OS cells and observed similar results after olaparib treatment, such as the suppression of NAD^+^-dependent mtPAR induction (Supplementary Fig. 3b). Olaparib inhibited PARP1, suggesting that the proteins are present in mitochondria, and so we next sought to determine whether mitochondrial PARylation was mediated by mtPARP1.

We tested the dependence of mitochondrial PARylation on mtPARP1 using purified mitochondria from PARP1-KO cells (Fig. 2b). Unlike mitochondria from WT cells, we observed no NAD^+^-mediated stimulation or olaparib-mediated repression of mitochondrial PAR in PARP1-knockout cells, confirming that PARP1 is the major mediator of mitochondrial PARylation.

We next examined the mitochondrial PAR response to oxidative stress in more detail. It is possible that a high concentration of H_2_O_2_ (5 mM) can damage the structural integrity of mitochondria, leading to unresponsive PARylation (Fig. 2a, 1st vs. 4th lane). To rule out this possibility, we used various concentrations of H_2_O_2_ (ranging from 0 to 500 µM, Fig. 2c). Even at lower concentrations of H_2_O_2_, there was no significant induction of mitochondrial PARylation (Fig. 2c, 1st–4th lanes), while NAD^+^-induced PAR induction was present (Fig. 2c, 5th–8th lanes). As this response to H_2_O_2_ was unexpected, we tested the DNA damaging agent menadione, which generates ROS through redox cycling^31^ and induces cell death in a PARP1-dependent manner^32^. Consistent with the H_2_O_2_ results, no mitochondrial PARylation was observed following menadione treatment (Fig. 2d, 1st–4th lanes). Menadione is known to trigger PARP1 activation in the nucleus^32^. Thus, in isolated mitochondria, the H_2_O_2_ and menadione results suggest that the activation of mtPARP1 is distinctly different from nuclear PARP1-mediated PARylation.

While NAD^+^ is the best-characterized substrate for PARP1-mediated PARylation, we next examined whether other NAD^+^ precursors could lead to alterations in mitochondrial PARylation. NAD^+^ precursors, such as nicotinamide (NAM), nicotinamide mononucleotide (NMN), and nicotinamide riboside (NR), were added to isolated mitochondria. NAM is a precursor of NAD^+^ in the salvage pathway and is an inhibitor of PARP1 in vitro^33^. None of these NAD^+^ precursors affected mitochondrial PARylation, but NAD^+^ itself did (Fig. 2e, f). These data suggest that NAD^+^ availability is the rate-limiting step for mtPARP1-mediated mitochondrial PARylation.

### Evaluating mitochondrial PARylation by ELTA

**Fig. 3:**
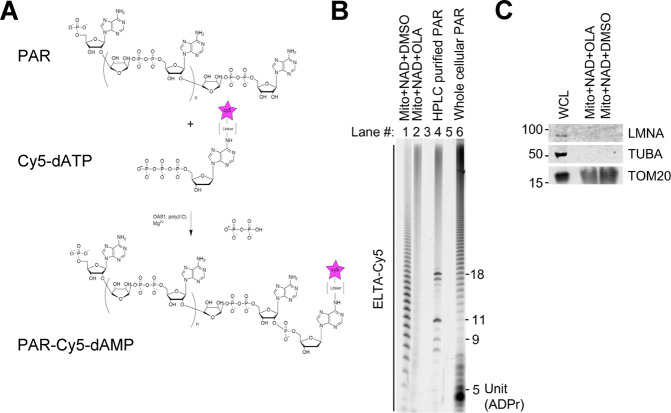
ELTA evaluation of mitochondrial PAR. **a** 2’-OH termini of ADP-ribose are labeled by OAS1-mediated oligomerization of Cy5-dATP; see Materials and Methods for details. **b** 15% urea-PAGE analysis of the ELTA labeling reaction of ADP-ribose isolated from mitochondrial reactions. Lanes #1 and 2 contain ELTA labeling of purified mitochondria that were incubated with NAD^+^ with/without 5 µM olaparib, while lane #6 is from whole cells. HPLC-purified PAR was loaded onto a PAR ladder (lane #4). **c** Corresponding lysates from mitochondria/cells in lanes #1, 2 and 5 were analyzed by western blotting with the indicated antibodies.

To further evaluate mitochondrial PARylation, we used ELTA to label ADP-ribose polymers isolated from mitochondria using the enzymes 2’-5’-oligoadenylate synthetase 1 (OAS1) and Cy5-dATP (Fig. 3a)^13^. PAR polymers were detached, purified, and enriched from the mitochondrial fractions (+NAD/+NAD+ olaparib), subjected to OAS1-mediated ELTA and labeled with Cy5-dATP for visualization on a sequencing gel (Fig. 3b). We used HPLC-purified PAR with predefined chain lengths as a marker (Fig. 3b, 4th lane). PAR from HeLa cell lysates was used as an additional reference, which showed various chain lengths (Fig. 3b, 6th lane). ELTA-labeled mitochondrial PARylation exhibited patterns similar to those of cellular PARylation, showing abundant polymers that were more than ~5 ADP-ribose moieties (Fig. 3b, 1st lane). Consistent with the earlier findings (Fig. 2a), olaparib treatment significantly abolished the PAR patterns (Fig. 3b, 2nd lane). Again, the purity of the mitochondrial samples that were subjected to ELTA was evaluated and shown to be free of cytosolic and nuclear contamination (Fig. 3c). Together with the antibody detection-based results (Fig. 2), these data indicate that PAR can be produced in isolated mitochondria in an NAD^+^-dependent and PARP1 inhibitor-sensitive manner.

### PARP1 induces mitochondrial nucleoid PAR accumulation

Having established the presence of mtPARP1 and NAD^+^-dependent mitochondrial PARylation, we sought to determine whether the observed PARylation occurred on the mitochondrial nucleoid since nuclear PARP1 affects chromatin. MtDNA is not packaged into chromatin but is instead packaged into DNA‒protein assemblies called nucleoids, which are the segregating units of mtDNA and play important roles in cellular metabolism^34^.

**Fig. 4:**
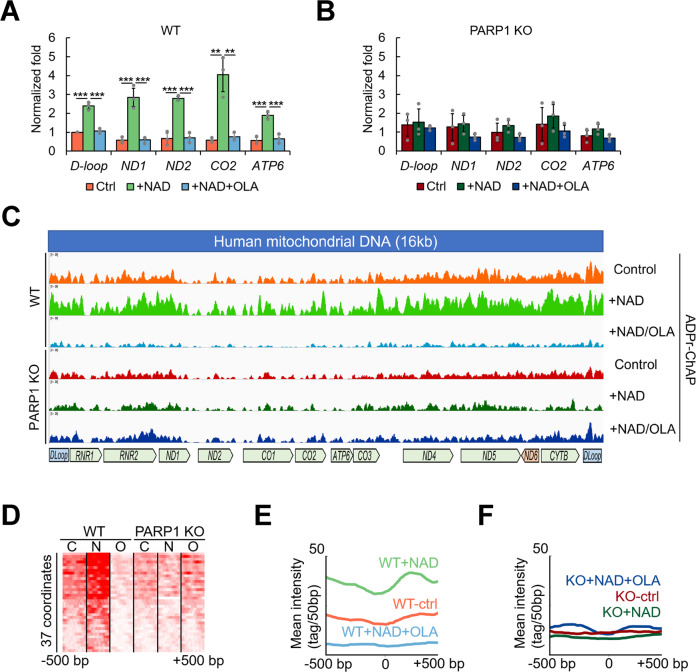
NAD^+^ stimulates PARP1-mediated PARylation of mitochondrial DNA. ADPr-ChAP was performed on purified mitochondria from WT (**a**) and PARP1-KO (**b**) cells that had been treated with 500 µM NAD^+^ with/without 5 µM olaparib for 30 min at 37 °C. After the reactions, mitochondrial samples were fixed for ADPr-ChAP. ADPr-ChAP purified mitochondrial DNA was subjected to real-time PCR using the indicated mtDNA-specific primers. Error bars represent the standard deviation. **c** ADPr-ChAP purified DNA from WT and PARP1-KO cells was sequenced, and the peak distribution on mtDNA was plotted by Integrative Genomics Viewer (IGV). **d** ADPr-ChIP sequencing reads of WT and PARP1-KO cells were aligned with seqMINER (Ver 1.3.3) on NC_012920 with 37 mitochondrial gene start sites as reference coordinates. Signal intensities are shown in the range of the mitochondrial gene start site ± 500 bp. C, control; N, + NAD; O, + NAD + olaparib. **e**, **f** Merged dataset profiles of aligned sequencing reads in D. One-way ANOVA was used to analyze the 7 groups followed by post hoc multiple pairwise comparisons using FDR correction to calculate p values for the pairwise comparisons. Error bars represent the standard deviation. *, <0.05; **, <0.01; ***, <0.001.

ADPr-ChAP, a newly developed method to detect nuclear PARylation^29^, was used to evaluate mitochondrial DNA PARylation. This is a robust and versatile method for analyzing chromatin ADP-ribosylation and is very similar to conventional ChIP; the major difference is the use of a PAR-specific-binding domain to precipitate ADP-ribosylated chromatin instead of an antibody^29^. We used a domain from the E3 ligase RNF146 that consists of two conserved tryptophans (W) and a glutamate (E) residue (WWE), which only binds to poly- but not mono-ADP-ribosylated proteins^35^. We adapted this technique to detect PARylation in isolated mitochondria to directly evaluate whether mitochondrial nucleoids were PARylated. The results of real-time qPCR analysis of the purified ADPr-ChAP samples for specific mitochondrial genes (normalized to input DNA) are shown. The results revealed significantly increased PARylation spanning the entire mitochondrial genome after NAD^+^ treatment (Fig. 4a). Consistent with the previous results (Fig. 2a and Supplementary Fig. 3c), NAD^+^-stimulated mitochondrial PARylation was suppressed by olaparib-mediated PARP1 inhibition (Fig. 4a, +NAD vs. +NAD+OLA). Moreover, mitochondria purified from PARP1-KO cells were not affected by NAD^+^ or olaparib treatment, confirming that PARP1 was responsible for the observed mitochondrial nucleoid PARylation (Fig. 4b).

ADPr-ChAP allows for mapping of the genome-wide location of ADP ribosylation and enables bioinformatic comparisons of ADP ribosylation with other chromatin modifications^17^, which is useful for understanding how ADP ribosylation regulates biologically important cellular processes^36^. To analyze the distribution of PAR on mitochondrial nucleoids, we subjected purified ADPr-ChAP products to DNA sequencing (Fig. 4c). Aligning the sequencing reads onto the mitochondrial genome, we generated a high-definition map of mitochondrial PARylation sites (Fig. 4c). Although PAR distribution showed dispersed enrichment over the mitochondrial genome (Fig. 4c, WT Ctrl), we observed a tilted distribution toward the D-loop region and sparsity around the ND2 locus. To compare the overall PAR distributions, we generated a heatmap of PAR signals on 37 mtDNA reference coordinates (13 proteins, 2 ribosomal, and 22 tRNAs) on mitochondrial gene start sites (Fig. 4d). Consistent with the real-time PCR data, WT cells showed significantly increased PAR signal intensity after NAD^+^ treatment, which was suppressed by olaparib treatment (WT C vs. N vs. O in Fig. 4d, e). Consistent with the earlier findings, NAD^+^ or olaparib treatment had minimal effects on mtDNA PARylation in PARP1-KO cells (PARP1-KO C vs. N vs. O in Fig. 4d, f).

### Biological consequences of PARP1 deficiency in mitochondria

Since persistent PARP1 activation causes intracellular NAD^+^ deprivation^5^, we hypothesized that PARP1 deficiency may cause changes in the intramitochondrial NAD^+^ metabolome. NAD^+^ metabolome analysis was performed on isolated mitochondria from WT and PARP1-KO cells^37^. Interestingly, there were minimal differences in NAD^+^ and its precursors, such as NR, NAM and NMN (data not shown). Additionally, there were minimal differences in PAR chain molecular building blocks, ADPR, and acetyl-CoA, which are mostly generated from pyruvate via NAD^+^-dependent processes^38^.

PARP1 negatively influences mitochondrial function and homeostasis via the depletion of cellular NAD^+^, and the deletion of PARP1 increases energy expenditure and protects against metabolic disease^7^. Using the Seahorse XF bioanalyzer, we evaluated mitochondrial function in WT and PARP1-KO HeLa cells. PARP1 deletion significantly increased the basal and maximal oxygen consumption rates (OCRs). The basal OCR increased by 11%, whereas maximal respiration was increased by 29%. Additionally, there was an increasing trend (*p* value 0.07) in oligomycin-sensitive respiration. (Supplementary Fig. 4).

Mitochondrial biogenesis, including mtDNA copy number, was increased in PARP1-depleted cells^10^. Thus, we examined whether mitochondria isolated from PARP1-KO cells had altered mtDNA copy numbers. Total cellular DNA was purified, and mitochondria-specific genes were estimated by real-time PCR and normalized to control nuclear DNA-specific targets^39^. We used the nuclear DNA-encoded genes *ATPAF* and *ING1* and the mtDNA-encoded genes *ND1* and *ND2* to calculate the mtDNA copy number. Both pairs of calculations resulted in consistent data, showing significantly increased mtDNA copy numbers in PARP1-KO cells, which was consistent with a prior publication^10^ (Supplementary Fig. 5).

### NAD+ treatment induces PARylation and regulates TFAM occupancy on the D-loop region

**Fig. 5:**
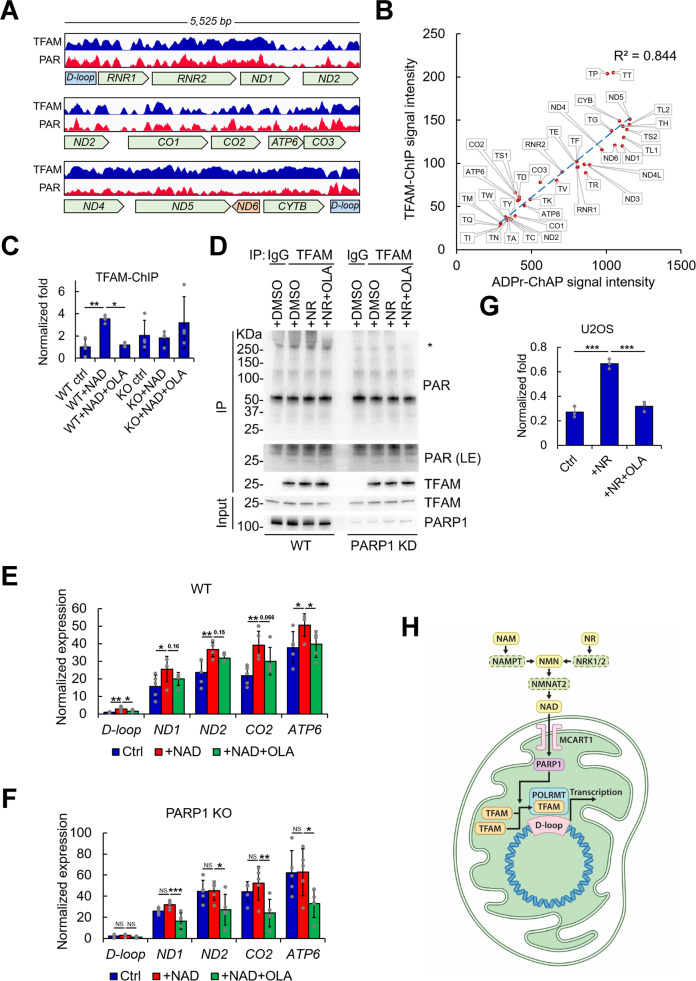
NAD+-induced PARylation stimulates mtDNA transcription. **a** TFAM-ChIP and ADPr-ChAP (PAR) were performed on mitochondria, and the sequencing reads were mapped onto the mitochondrial genome. Key mtDNA genes are shown. **b** ADPr-ChAP and TFAM-ChIP seq signal intensities of 37 mitochondrial genes at their respective gene start sites were calculated by seqMINER (Ver 1.3.3) and plotted. **c** HeLa WT and PARP1-KO cells were subjected to TFAM-ChIP. Two-way ANOVA was used to calculate the *p* value. **d** U2OS WT/PARP1-knockdown (KD) cells were treated with DMSO, 1 mM NR and 1 mM NR plus 5 µM olaparib for 24 h, followed by immunoprecipitation with normal rabbit IgG or TFAM antibodies. The immunoprecipitated products analyzed by western blotting with the indicated antibodies. **e**, **f** Isolated mitochondria from WT and PARP1-KO cells were treated with NAD + with/without olaparib for 30 min at 37 °C. Subsequently, mitochondrial samples were treated with RNase A to eliminate residual nuclear/cytoplasmic RNA. After the treatment, mitochondrial RNA was purified and reverse transcribed (*n* = 5). **g** U2OS cells were treated with NR with/without olaparib for 24 h. TFAM-ChIP was performed with purified mitochondria (*n* = 3). qPCR was performed with primers specific to the mtDNA D-loop locus. Error bars represent the standard deviation. *, <0.05; **, <0.01; ***, <0.001. **h** Schematic diagram of NAD^+^-induced PARylation stimulating mtDNA transcription. See text for details.

PARylation positively correlates with histone density in the nucleus because histones provide platforms for chromatin PARylation^29^. We hypothesized that the mitochondrial nucleoid may serve as an analogous platform for PAR. This process could involve TFAM, which plays a histone-like role in mitochondria by stabilizing mtDNA and packaging it into mitochondrial nucleoids^40,41^. To determine TFAM distribution on the mitochondrial genome, we performed TFAM-ChIP sequencing analysis. Consistent with a previous report^42^, TFAM binding did not show specific enrichment at its well-known binding sites in the D-loop (Fig. 5a, upper lane). Instead, it showed a broad and uneven distribution over the entire mitochondrial genome. This distribution showed striking similarities with mitochondrial nucleoid PAR signals across the mitochondrial genome (Fig. 5a, lower panel). The correlation of mitochondrial nucleoid PAR and TFAM distribution on mtDNA was further evaluated by plotting the 37 individual mitochondrial genes with their respective ADPr-ChAP and TFAM-ChIP seq signal intensities at the gene start sites (Fig. 5b). The trendline between the two signals showed a high correlation (*R*^2^ = 0.844), indicating that mitochondrial nucleoid PARylation and TFAM distribution were highly correlated. As shown in Fig. 5b, there were a few tRNA outliers (TP and TT) close to the D-loop region; however, to the reasons for this phenomenon are presently unclear.

TFAM binding to the D-loop region is a critical step in mtDNA transcription^43–46^. Thus, we used TFAM-ChIP to determine whether NAD^+^ treatment-induced PAR accumulation affected TFAM recruitment to the D-loop region. NAD^+^ treatment of purified mitochondria significantly stimulated TFAM recruitment, while olaparib treatment abolished this effect (Fig. 5c, WT ctrl vs. WT+NAD vs. WT+NAD+Ola). Consistent with the earlier observations, mitochondria isolated from PARP1-KO cells showed little response to NAD^+^ or olaparib treatment (Fig. 5c, KO ctrl vs. KO+NAD vs. KO+NAD+Ola). These results suggest that NAD^+^-mediated PARylation by mtPARP1 is critically involved in TFAM binding to the mitochondrial D-loop. Since we observed high overlap of TFAM and PAR signals in the mitochondrial genome (Fig. 5a, b), we examined whether NAD^+^ treatment affected PAR accumulation directly on TFAM. NR treatment induces mitochondrial NAD^+^ production in cells^47^, and so WT/PARP1-knockdown U2OS cells were treated with NR with or without olaparib for 24 h, followed by TFAM-immunoprecipitation; subsequently, PAR was analyzed by western blotting to determine TFAM PARylation (Fig. 5d). Examining TFAM IP products with the PAR antibody showed a PARylation band at a high molecular weight after NR treatment (Fig. 5d, asterisk). This PARylation was suppressed by olaparib (Fig. 5d, WT, +NR+Ola). Moreover, PARP1 knockdown suppressed NR treatment-induced high-molecular-weight PARylation (Fig. 5d, PARP1 knockdown). We were also able to detect a faint band by PAR IB at the same molecular weight as TFAM (Fig. 5d, PAR long exposure). However, we did not observe a shifted band above the original molecular weight of TFAM, nor did we observe any changes in this product after PARP1 knockdown treatment with NR or olaparib. Although it is unclear whether the high molecular weight product (asterisk) contains PARylated TFAM, these observations were consistent with the earlier results showing increased TFAM interactions with NAD^+^-induced PARylation, which as almost completely abolished by PARP1 knockdown or olaparib treatment (Fig. 5d, WT vs. PARP1 knockdown and +NR vs. +NR+OLA). Taken together with the TFAM-ChIP data, it is likely that PARylation of the mitochondrial nucleoid induces TFAM recruitment.

Since we found that mitochondrial nucleoid PARylation induces TFAM recruitment and that TFAM binding to the D-loop region is a critical step in mtDNA transcription, we examined whether mtDNA transcription was affected by mitochondrial nucleoid PARylation. Notably, NAD^+^ treatment significantly increased mtDNA transcription (Fig. 5e, WT ctrl vs. WT+NAD), and olaparib treatment abolished NAD^+^ treatment-induced mitochondrial transcription (WT+NAD vs. WT+NAD+OLA). Moreover, in PARP1-KO mitochondria, NAD^+^ treatment had no effect on transcription status (Fig. 5f, KO ctrl vs. KO+NAD). To determine whether NAD^+^-induced TFAM recruitment was restricted to HeLa cells, we examined whether NAD^+^ could induce TFAM recruitment to mtDNA in U2OS cells. The TFAM-ChIP results from U2OS cells showed that NR treatment significantly increased TFAM recruitment to the mtDNA D-loop region in these cells (Fig. 5g, Ctrl vs. +NR). Consistent with the ex vivo experiments using purified mitochondria, olaparib treatment suppressed TFAM recruitment in response to NR treatment (Fig. 5g, +NR vs. +NR+OLA). Taken together, these data suggest that mtPARP1-induced mitochondrial nucleoid PARylation mediates TFAM recruitment to the D-loop to regulate mtDNA transcription (Fig. 5h).

## Discussion

Several studies have reported the presence of mtPARP1 and its ADP-ribosylation activity^10,48–51^. However, there has been a lack of direct evidence, which has led to much skepticism about mtPARP1. Some studies claim that mitochondria are completely devoid of PARP1, that PARylated proteins enter mitochondria^52^ or that mitochondria harbor enzymes other than PARP1 with ADP-ribosyltransferase activity^11^. On the other hand, some studies report the involvement of PARP1 in mtDNA metabolism, demonstrating direct interactions between PARP1 and mtDNA for mtDNA maintenance^9^ and repair^53^. In the present study, we investigated the presence of mtPARP1 and directly observed mitochondrial PARP1 and its activity in isolated mitochondria using methodologies including western blotting, ELTA, ChIP, and ADPr-ChAP seq. We tried to avoid imaging-based analysis to evaluate mitochondrial PARP1 localization because the different antibody qualities and fixation conditions result in experimental artifacts^54^. Instead, we used mitochondrial isolation with stringent quality control and mitochondrial DNA-specific ChIP and ADPr-ChAP. In fact, the mitochondrial isolation step has been often omitted during mitochondrial ChIP because it is followed by mtDNA-specific PCR analysis. Thus, the highly specific nature of ChIP and ADPr-ChAP make them ideal tools to investigate mtPARP1. PARP1 plays critical roles in DNA damage signaling and DNA repair in the nucleus. It has also been reported that mtPARP1 is involved in the maintenance of mtDNA integrity^10,49^ and repair^55,56^, suggesting a close interaction between PARP1 and mtDNA. We observed direct PARP1 binding to mtDNA through its DNA-binding domain (Fig. 1e, f). Moreover, we were the first to show a detailed PARylation map of mtDNA (Fig. 4c).

There is much interest and debate about the presence and nature of mitochondrial PAR. Hopp et al. recently reported the absence of mitochondrial PAR by showing a lack of mitochondrial signal after cellular anti-PAR immunofluorescence (IF) staining^11^. However, upon close examination of the published IF data, their results indicate some mitochondrial PAR localization, although the signals were weaker than the nuclear PAR signal. Moreover, the authors used a mask minimally spaced 1.6-26 μm from the nuclear periphery for their quantitative image-based cytometry (QIBC) analysis, which may have excluded perinuclear mitochondria. Additionally, in support of mitochondrial poly-ART, PARylation of many mitochondrial proteins has been identified in previously published proteomic studies^57^, including the finding of diverse PARylated proteins in mitochondrial extracts of HeLa cells by LC-MS/MS using isobaric tandem mass tag (TMT) labeling^58^. Thus, the different conclusions on mtPAR may be related to the different approaches used and the cells or tissues studied. In the current study, we cross-confirmed the presence of mitochondrial PARylation by western blotting, ELTA, and ADPr-ChAP.

Oxidative stress is a strong stimulator of nuclear chromatin PARylation. MtDNA is reported to be more sensitive to oxidative damage than nuclear DNA^59,60^. However, we observed that H_2_O_2_ or menadione at concentrations that induce mtDNA damage^61–63^ did not induce mitochondrial PAR in isolated mitochondria (Fig. 2c, d). This finding suggests that direct oxidative stress-mediated mtDNA damage to isolated mitochondria does not stimulate mitochondrial nucleoid PARylation. A previous study showed that mtPARP1 but not nuclear PARP1 exerted a negative effect on oxidative damage-induced mtDNA repair^10^. Another recent study showed decreased mitochondrial ADP ribosylation in cells treated with H_2_O_2_^11^. It is likely that the temporal reduction in mitochondrial NAD^+^ in response to nuclear PARylation causes NAD^+^ deprivation and low PARylation activity. This finding is consistent with the findings that the addition of NAD+ to isolated mitochondria was required for robust PARylation (Fig. 2c), suggesting that mitochondrial PARylation is NAD^+^ dependent.

NAM, NMN, and NR can be supplied by food intake or endogenous production and effectively enhance NAD^+^ biosynthesis. In mammals, NMN is synthesized from NAM by the rate-limiting enzyme nicotinamide phosphoribosyltransferase (NAMPT). NMN is also synthesized from NR via an NR kinase (NRK)-mediated phosphorylation reaction. NMN is then converted into NAD^+^ by NMN adenylyltransferases (NMNATs), and NMNAT3 is the only NAD^+^ biosynthetic enzyme localized in the mitochondrial matrix^64^. It is still an open question whether NAM, NMN and NR can enter the mitochondria directly. Interestingly, none of these compounds stimulated mitochondrial PARylation under the experimental conditions (Fig. 2e, f). This finding suggests a lack of NAM, NMN and NR transporter systems or the absence of an NAD^+^ biosynthetic mechanism for these compounds within mitochondria. We recognize that the recently identified mitochondrial NAD^+^ transporter MCART1/SLC25A51 likely mediated NAD^+^ entry in our experiments^65,66^. MCART1/SL22A51 is an inner mitochondrial membrane protein that drives mitochondrial NAD^+^ uptake, which is required for mitochondrial respiration.

After oxidative stress, chromatin PAR levels are highest on heterochromatic sites and depleted at active promoters, correlating with local histone abundance^29^. Likewise, we found a high correlation between mitochondrial nucleoid PAR and TFAM occupancy on mtDNA (Fig. 5a, b). Moreover, TFAM recruitment to the mtDNA D-loop locus was induced by PARylation (Fig. 5c), which influenced mtDNA transcription (Fig. 5e).

TFAM is a multifunctional DNA-binding protein that is essential for transcriptional activation and mtDNA organization^67–70^. Some posttranslational modifications of TFAM that regulate its DNA binding were suggested to be involved in the regulation of mtDNA transcription^71–73^. Although TFAM is a known substrate for PARP1^74,75^, we could not conclude that TFAM PARylation was dependent on PARP1 in our experimental settings (Fig. 5d). It is also possible that the PARylation of other mitochondrial nucleoid components occurred in the study. Nevertheless, we observed that PARylation regulated TFAM occupancy on mtDNA, suggesting that mitochondrial PAR is an important regulator of mtDNA transcription. In addition, we showed that mitochondrial PARylation was induced in an NAD^+^-dependent manner (Fig. 2), suggesting that mtPARP1 may act as an NAD^+^ sensor and use mitochondrial nucleoid PARylation as a signal transduction pathway to regulate mtDNA transcription. Thus, mtPARP1 may serve as a protein scaffold that mediates TFAM occupancy of mtDNA. Notably, mtDNA-encoded electron transport chain (ETC) gene transcription is regulated by the transcription of nuclear genes encoding components of the ETC such that there is not an overabundance of individual components of the ETC. Whether NAD^+^ has the capacity to also modulate the transcription of nuclear-encoded ETC genes will require further research.

We did not find significant NAD^+^ metabolome changes in PARP1-KO cells (data not shown). Presumably, the NAD^+^ pool in PARP1-KO cells is readily available for other NAD^+^ consumers, similar to intracellular sirtuin activity. Although the effect of PARP1 inhibition on SIRT3 is controversial^7,76^, it has been reported that reduced activity of SIRT3 correlates with decreased mtDNA binding activity of TFAM and results in reduced mtDNA transcription^77^ and copy numbers^78^. SIRT3 overexpression increases mtDNA copy number and alters cellular metabolism^79^. These findings are consistent with the observations that PARP1-KO cells have improved mitochondrial respiration (Supplementary Fig. 4), as well as increased mtDNA copy numbers (Supplementary Fig. 5), and we hypothesize that SIRT3 can promote these changes.

We observed a decrease in mtDNA copy number in WT cells (Supplementary Fig. 5), and PARP1 activation induced by NAD^+^ increased RNA transcription (Fig. 5e) in mitochondria from WT cells. These may seem to be conflicting events because RNA transcripts are the primers for mtDNA replication. In fact, we did observe higher basal transcription levels in PARP1-KO cells than in WT cells (Fig. 5e vs. f, Controls). However, PARP1-KO cells were unresponsive to NAD^+^ treatment, while RNA transcription was significantly stimulated in WT cells. Thus, we hypothesize that the short duration of NAD^+^ treatment under ex vivo conditions (~30 min) was insufficient to complete mtDNA replication for copy number determination, as the mammalian mtDNA replication time is estimated to be approximately 2 h^80^. We showed that RNA transcription levels in PARP1-KO cells responded to olaparib treatment. This finding suggests that another PARP (PARP2/3) and/or the recently identified mitochondrial ADP-ribosyltransferase Neuralized-like protein 4 (NEURL4)^81^ may be involved in steady-state nucleoid PARylation or could be an off-target effect of olaparib.

Interestingly, it has been shown that a portion of bacterial, yeast, and human RNA undergoes NAD^+^ capping instead of 5’ methyl guanosine capping by mitochondrial RNA polymerase (mtRNAP)^82–86^. The efficiency of this process is determined by intracellular NAD^+^ levels, and 5’ NAD^+^ capping of RNA is shown to be abundant in human mitochondrial RNA and may modulate RNA stability and translatability^87^. Consistent with this, the results may indicate a mechanism by which local mitochondrial NAD^+^ concentrations can regulate mtDNA transcription through TFAM recruitment. This novel pathway may serve as a sensor and actuator by coupling cellular metabolism to mitochondrial gene expression. The data showed that NAD^+^ treatment was not sufficient to induce mitochondrial transcription in mitochondria lacking PARP1 (Fig. 5f), suggesting that mtPARP1-mediated TFAM recruitment was important for NAD^+^ sensing and the initiation of mtDNA transcription.

Overall, we provide new and direct evidence of PARP1 in mitochondria and show that PARP1 binds directly to mtDNA and induces NAD^+^-dependent mitochondrial nucleoid PARylation. We also show that mitochondrial nucleoid PARylation regulates the recruitment of TFAM to stimulate mtDNA transcription (Fig. 5h). These data identify a novel mtPARP1-NAD^+^-PAR axis involved in the regulation of mitochondrial homeostasis and may provide insight into the molecular mechanisms of mitochondrial dysfunction that underlie aging and may facilitate the development of novel interventional strategies for human health span extension.

## Supplementary information

Supplementary Figures

Supplementary table 1

## Data Availability

The accession number for the raw and processed ChIP sequencing and ADPr-ChAP sequencing data reported in this paper is GEO: GSE182533.
