# Supplementary material for: Mitochondrial PARP1 regulates NAD+-dependent poly ADP-ribosylation of mitochondrial nucleoids
Source: Exp Mol Med. 2022 Dec 6;54(12):2135–47. doi: 10.1038/s12276-022-00894-x (PMC9794712; doi:10.1038/s12276-022-00894-x)
Supplement: Supplementary file 1 — Supplementary Figures [file 12276_2022_894_MOESM1_ESM.pdf]

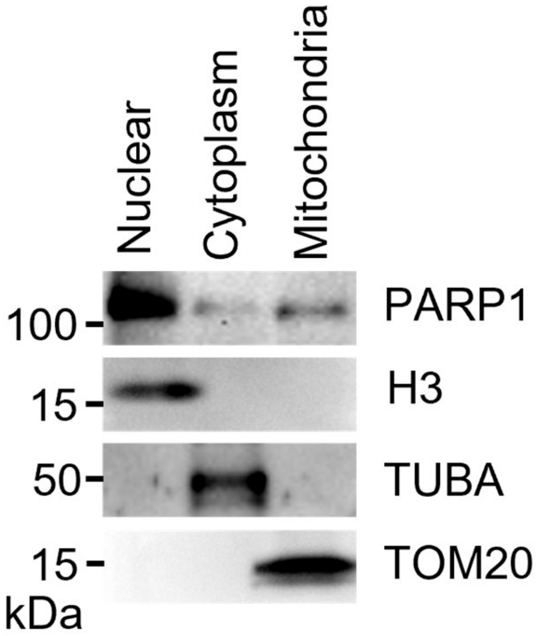

Nuclear
Cytoplasm
Mitochondria
100
15
50
15
kDa
PARP1
H3
TUBA
TOM20

Supplementary Fig. 1. Subcellular distribution of PARP1

Western blot evaluation of PARP1 in subcellular fractions from HeLa WT cells. Purified nuclear, cytoplasm, and mitochondria were probed for western blot with indicated antibodies.

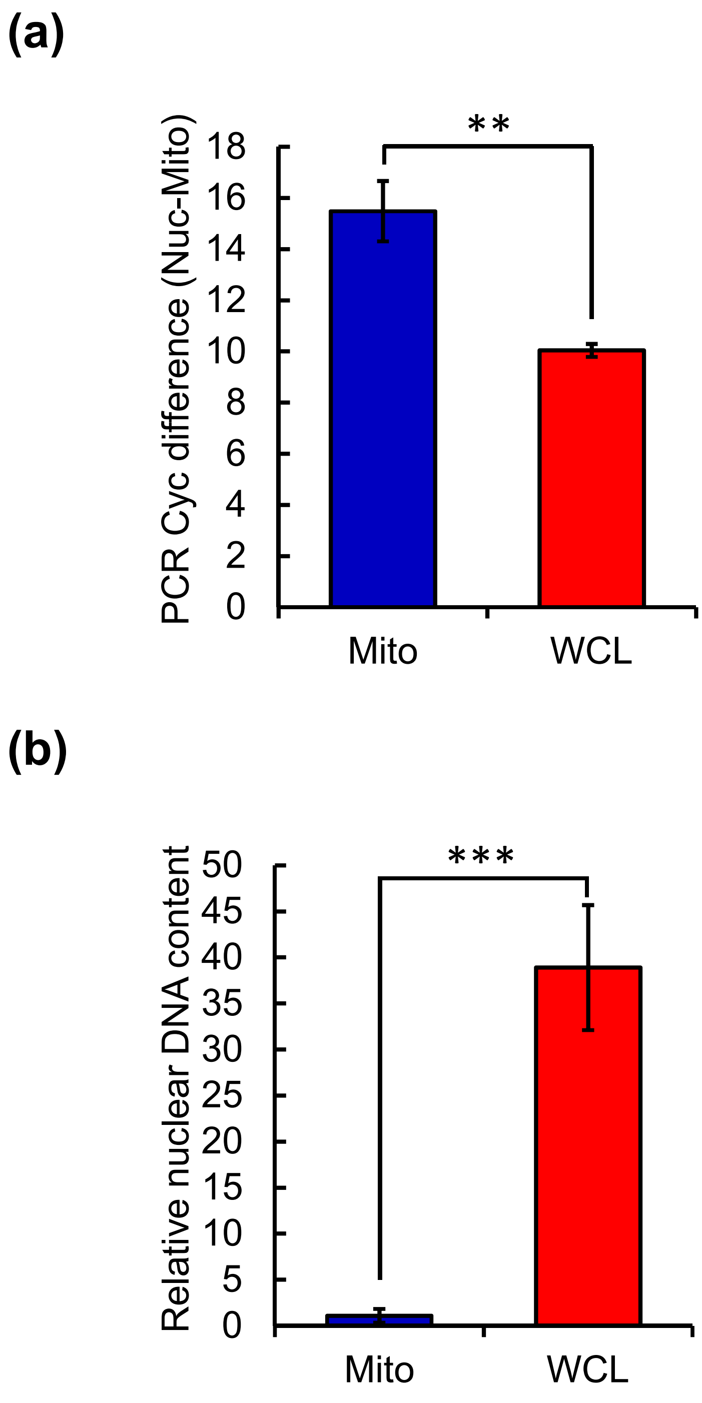
(a)
PCR Cyc difference (Nuc-Mito)
**
18
16
14
12
10
8
6
4
2
0
Mito
WCL
(b)
Relative nuclear DNA content
***
50
45
40
35
30
25
20
15
10
5
0
Mito
WCL

Supplementary Fig. 2. Estimation of nuclear contamination by realtime PCR.

Whole cell lysates (WCL) and purified mitochondria from WT/PARP1 KO HeLa were prepared for ChIP/ChAP. Purified DNAs were evaluated for nuclear contamination using five mitochondria and three nuclear specific genes (n=3). (a) Average PCR CT value differences between average mitochondrial and nuclear genes were compared. (b) Comparison of nuclear DNA contamination in purified mitochondria versus WCL. Relative nuclear DNA contaminations are calculated by normalization with the amount of nuclear DNA in purified mitochondria. Error bars represent standard deviation. **, <0.01; ***, <0.001.

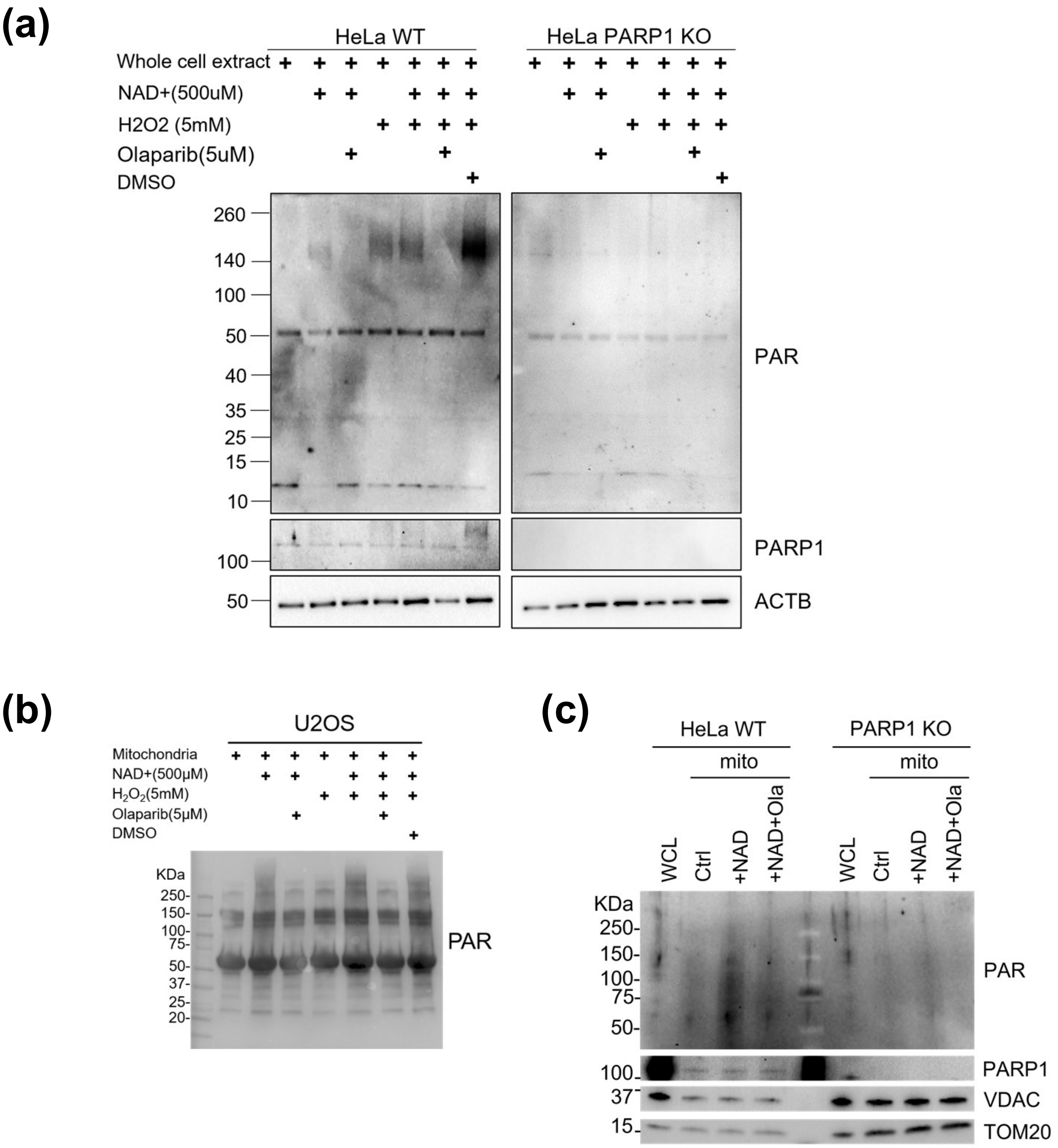
(a)
HeLa WT
HeLa PARP1 KO
Whole cell extract
NAD+(500uM)
H2O2 (5mM)
Olaparib(5uM)
DMSO
260
140
100
50
40
35
25
15
10
PAR
PARP1
100
50
ACTB
(b)
U2OS
Mitochondria
NAD+(500μM)
H2O2(5mM)
Olaparib(5μM)
DMSO
KDa
250-
150-
100-
75-
50-
37-
25-
20-
PAR
(c)
HeLa WT
PARP1 KO
mito
mito
WCL
Ctrl
+NAD
+NAD+Ola
WCL
Ctrl
+NAD
+NAD+Ola
KDa
250-
150-
100-
75-
50-
PAR
100
PARP1
37
VDAC
15
TOM20

Supplementary Fig. 3. NAD^+ stimulates mitochondrial PAR by PARP1

(a) HeLa WT and PARP1 KO cells were treated with were treated with indicated combinations of reagents for 30 minutes at 37 °C and whole cell extracts were subjected for western blot, probed with indicated antibodies. (b) NAD^+ stimulates mitochondrial PAR by PARP1. Purified mitochondria from U2OS cells were treated with indicated combinations of reagents for 30 minutes at 37 °C and 1.3ug of mitochondrial samples were subjected for western blot, probed with PAR specific antibody. (c) Western blot evaluation of purified mitochondria for ChIP/ChAP. Isolated mitochondria from WT/KO cells were treated with NAD^+ with/without olaparib for 30 minutes at 37 °C. After reaction, mitochondrial samples were resuspended in ChIP/ChAP buffer B. mtDNAs were digested, DNA concentrations were measured and normalized. Same amount mtDNA containing mitochondrial samples were probed for western blot with indicated antibodies. Whole cell lysates for each cell were loaded as a control. Error bars represent SD.

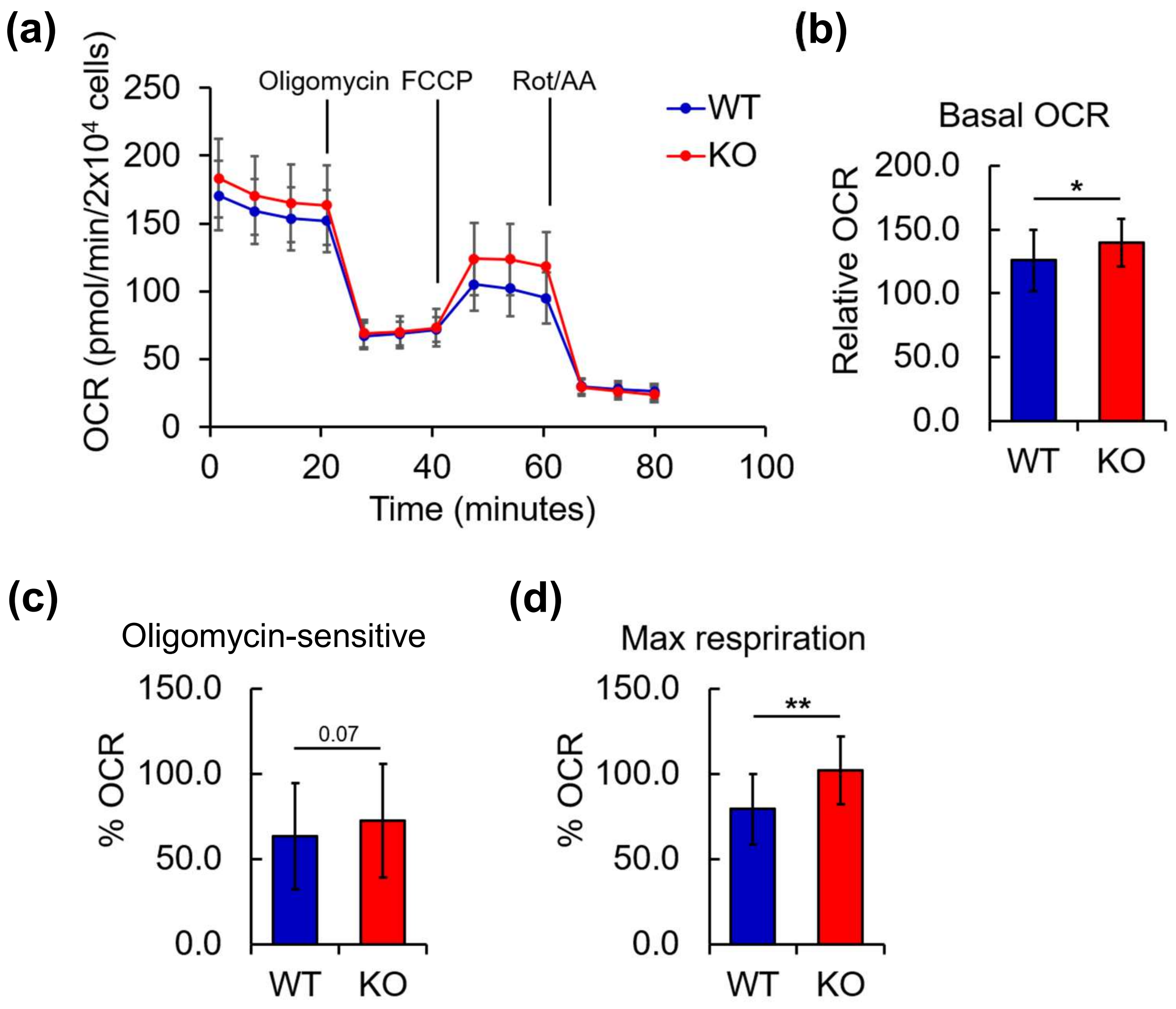
(a)
OCR (pmol/min/2x10⁴ cells)
Oligomycin
FCCP
Rot/AA
WT
KO
250
200
150
100
50
0
0
20
40
60
80
100
Time (minutes)
(b)
Basal OCR
Relative OCR
200.0
150.0
100.0
50.0
0.0
*
WT
KO
(c)
Oligomycin-sensitive
% OCR
150.0
100.0
50.0
0.0
0.07
WT
KO
(d)
Max respriration
% OCR
150.0
100.0
50.0
0.0
**
WT
KO

Supplementary Fig. 4. Seahorse extracellular flux analysis of intact cells

OCR was measured in WT and KO HeLa cells using Seahorse extracellular flux analysis, according to Materials and Methods (n=5). (a) Seahorse extracellular flux analysis representative trace. (b) Basal respiration was calculated and normalized to the number of cells in each well. (c) and (d) Oligomycin-sensitive respiration and Maximal respiration capacity are measured via the calculation of relative changes between FCCP and Rotenone/Antimycin A injection. Error bars represent SD. *,<0.05; **, <0.01.

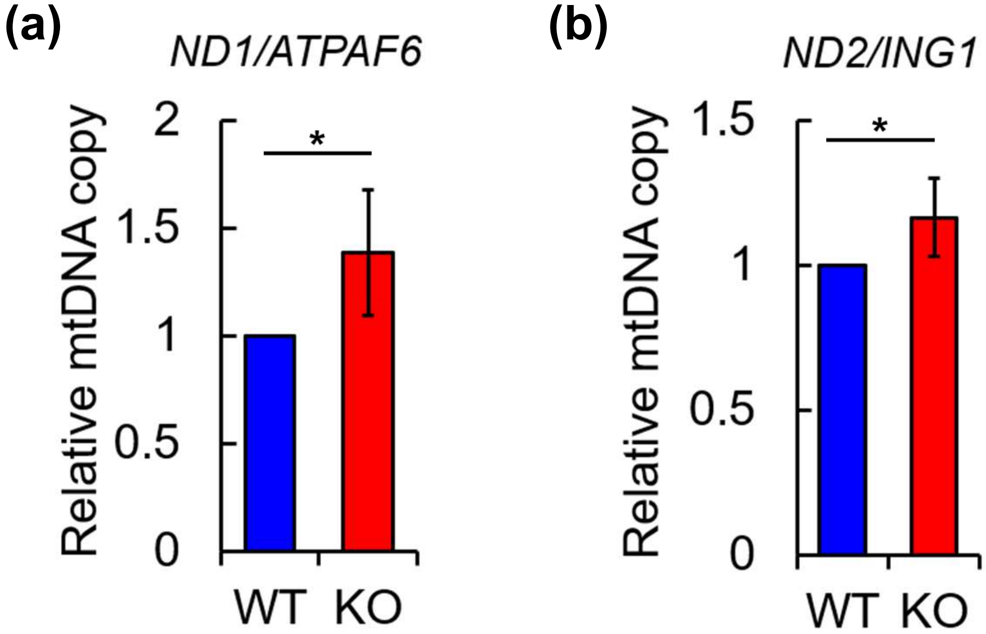

(a)
ND1/ATPAF6
Relative mtDNA copy
2
1.5
1
0.5
0
*
WT
KO
(b)
ND2/ING1
Relative mtDNA copy
1.5
1
0.5
0
*
WT
KO

Supplementary Fig. 5. mtDNA copy number assessment

Total cellular DNA were purified from WT/KO cells, mitochondria specific genes were estimated by realtime PCR and normalized with control nuclear DNA specific targets.

Two nuclear DNA encoded genes, (a) *ATPAF* and (b) *ING1* were used to normalize mtDNA encoded genes *ND1* and *ND2,* respectively, to calculate mtDNA copy number (n=6). Error bars represent SD. *,<0.05.
